# Arc and forearc rifting in the Tyrrhenian subduction system

**DOI:** 10.1038/s41598-022-08562-w

**Published:** 2022-03-18

**Authors:** M. Corradino, A. Balazs, C. Faccenna, F. Pepe

**Affiliations:** 1 Department of Earth and Marine Sciences, University of Palermo, Via Archirafi, 22, 90123 Palermo, Italy; 2 Department of Earth Sciences, ETH Zürich, Zürich, Switzerland; 3 GFZ German Research Centre for Geosciences, Potsdam, Germany; 4 Department of Sciences, Università Roma TRE, Rome, Italy

**Keywords:** Solid Earth sciences, Geodynamics

## Abstract

The evolution of forearc and backarc domains is usually treated separately, as they are separated by a volcanic arc. We analyse their spatial and temporal relationships in the Tyrrhenian subduction system, using seismic profiles and numerical modelling. A volcanic arc, which included the Marsili volcano, was involved in arc-rifting during the Pliocene. This process led to the formation of an oceanic backarc basin (~ 1.8 Ma) to the west of the Marsili volcano. The eastern region corresponded to the forearc domain, floored by serpentinised mantle. Here, a new volcanic arc formed at ~ 1 Ma, marking the onset of the forearc-rifting. This work highlights that fluids and melts induce weakening of the volcanic arc region and drive the arc-rifting that led to the backarc basin formation. Later, the slab rollback causes the trench-ward migration of volcanism that led to the forearc- rifting under the control of fluids released from the downgoing plate.

## Introduction

Forearc basin represents a recurrent feature over the subduction zone and forms between the arc and the trench along with the overriding plate. Its evolution is punctuated by phases of subsidence and uplift, and characterised by compressional and extensional episodes. The mechanisms of forearc basin formation are poorly understood, and probably reflect the behaviour of megathrust and the overall response of the upper plate to slab rollback and trench retreat^1–4^. On the other side of the volcanic arc, the backarc basin form when the subduction rate exceeds the plate convergence rate^5–7^, leading to lithospheric stretching and locally to continental break-up, mantle exhumation or seafloor spreading^8^.

The evolution of backarc and forearc basins is usually treated separately, as the volcanic arc represents a clear barrier between them. Their spatial extension and temporal evolution are associated with processes occurring in a continuously evolving subduction zone^9–11^, such as the trench-ward migration of volcanism and the oceanic crust formation.

The Mediterranean represents an outstanding example of this interaction. The retreat of the subduction zones in the region produced extensional backarc basins^12–14^ like the Algerian-Provenca, Tyrrhenian, Aegean or the Pannonian, but also smaller basins, like the Transylvanian basin, the Cretan Sea basin, the Marsili basin, and the Gibraltar basin.

Here, we will reconstruct the origin of one of those basins, the Marsili basin in the Tyrrhenian Sea (Central Mediterranean), using seismic reflection profiles coupled with literature data. We constrain the crustal structure of the basin revealing the different seismic characteristics on both sides. We show that the oceanic backarc basin corresponds to the area west of the Marsili volcano only, and it results from the arc-rifting process that affected an old (Pliocene) volcanic arc. The eastern side of the Marsili basin, commonly interpreted as a part of the backarc basin, is instead a forearc region floored by serpentinised mantle. This domain is later involved in an extensional process responsible for forming a new volcanic arc and the onset of a backarc basin behind it. Using numerical modelling, we show that the transition from forearc to backarc represents a common dynamical evolutionary scenario during trench rollback in the presence of fluids released from the slab. Our results bring then insight into the upper plate evolution during slab rollback.

### Tectonic background

The evolution of the western Mediterranean basin is produced by the subduction and retreat of the Adriatic‐Ionian plate beneath Eurasia since Oligocene times^15^. In Central Mediterranean, this process first led to the opening of the Liguro-Provençal basin^16,17^ and then of the Tyrrhenian basin^18–20^. At place, like on the Gulf of Lyon, the extension also caused lower crustal and mantle exhumation that now marks the transition towards the oceanic crust^21^. In the Tyrrhenian basin, backarc extension led to moderate lithospheric and crustal thinning along the basin margins and two spreading centres, ca. 80 km apart, the Vavilov (4.3–4.1 to 2.6 Ma) and Marsili basins (Late (?) Pliocene to ~ 1.87 Ma)^20^. Crustal thinning also produced mantle exhumation, magmatic accretion (Magnaghi and Vavilov basins)^22^ and intrusion of mid-ocean ridge basalts^23^. OIB-MORB type basalts and Island Arc Basalts (IAB) formed in the Vavilov and Marsili basins, respectively^24^.

**Figure 1:**
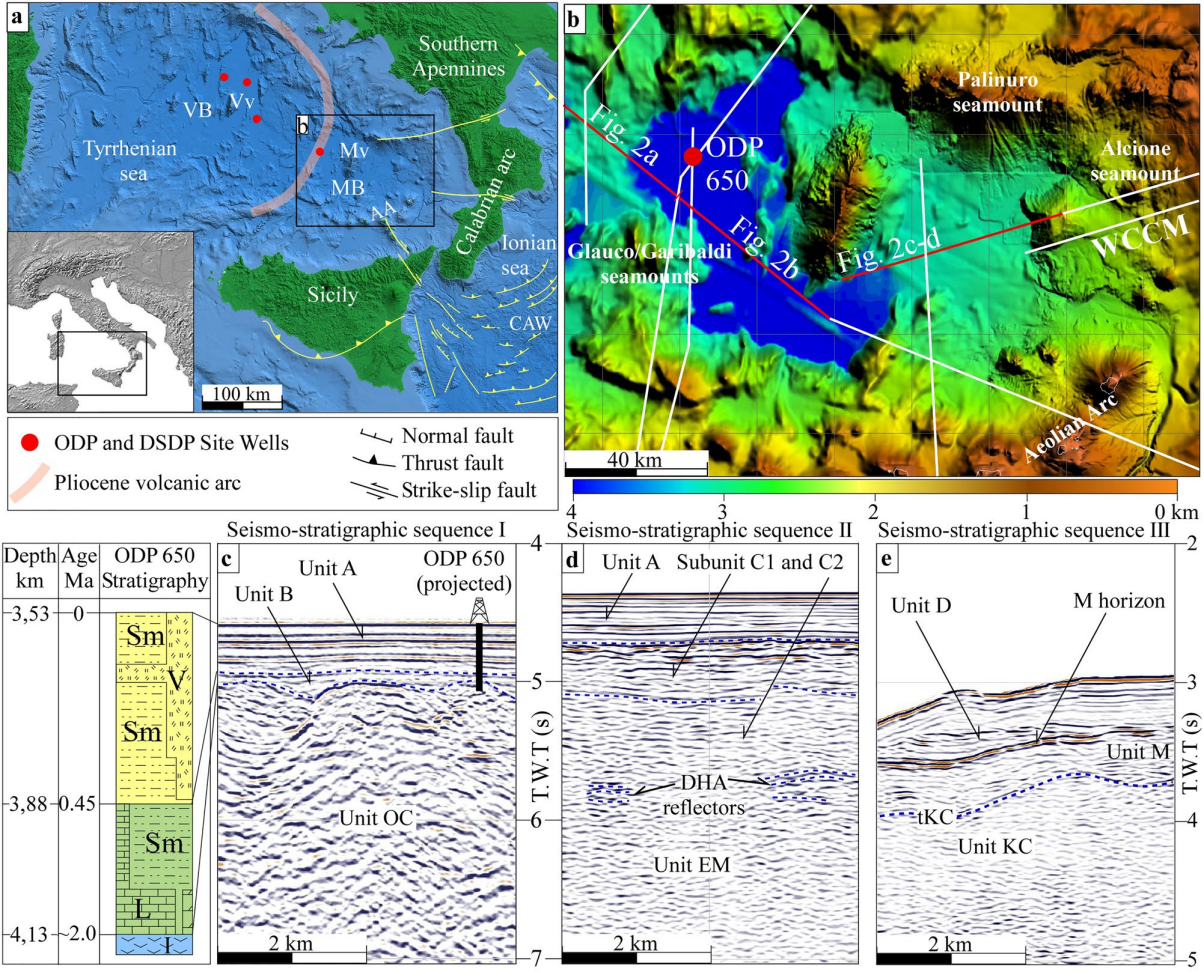
(**a**) Schematic tectonic map of the Tyrrhenian‐Ionian subduction system. AA: Aelonian Arc, CAW: Calabrian accretionary wedge, MB: Marsili basin, Mv: Marsili volcano VB: Vavilov basin, Vv: Vavilov volcano. Inset shows the location of the area. (**b**) Morphobathymetric map of the Marsili basin and surroundings. WCCM: western Calabrian continental margin. Red point indicates the location of the ODP 650 site well. Red and white lines are the traces of the seismic profiles used in this study. Red lines are the parts of the profiles shown in Fig. 2. The map was generated using the GeoSuite AllWorks software package (version 2021R2, https://www.geomarinesurveysystems.com/downloads/). (**c**, **d**, and **e**) Seismo-stratigraphic sequences I, II and III recognised on seismic profiles. T.W.T.: two‐way travel. Location of box c is displayed in Fig. 2a, and location of boxes d and e in Fig. 2c. Box c also shows the stratigraphic scheme of the units recognised on the ODP 650 site and their correlation with the seismo-stratigraphic units of sequence I. Sm: sandy mud, V: volcanoclastic rocks, L: limestone, I: igneous rocks. Correlation between seismic units and stratigraphy is based on seismic facies analysis and stratigraphic data from ODP Site 650.

The area between the Vavilov and Marsili basin hosts several volcanic seamounts (smt) representing a former Pliocene volcanic arc^25^ (Fig. 1a). The volcanic arc extends in the central Tyrrhenian Sea and includes the Anchise smt (5.2–3.6 Ma)^25^ in the south, the Glauco-Garibaldi smt in the centre, and the Pontine Islands (4.2–0.1 Ma)^26^ and buried calc-alkaline rocks of the Campanian Plain in the north^25,27,28^.

The Marsili volcano is a NNE-SSW elongated ridge located in the central part of the Marsili basin, and elevated ~ 3000 m from the abyssal plain (Fig. 1a and b). Rocks samples recovered from the Marsili volcano indicate that it is mainly composed of medium-K calc-alkaline basalts. High- evolved K andesites rocks samples were only retrieved from small cones on the summit axis zone^29^.

The Marsili volcano has been interpreted as the spreading ridge of the Marsili backarc basin^18,19,30,31^ or a superinflated ridge^32^.

## Results

### Seismic reflection data

We identified three seismo-stratigraphic sequences. Each consists of a vertical arrangement of seismic units, defined by their bounding unconformities and described based on their architecture and seismic characters (e.g., amplitude, lateral continuity, and frequency of internal reflectors).

**Figure 2:**
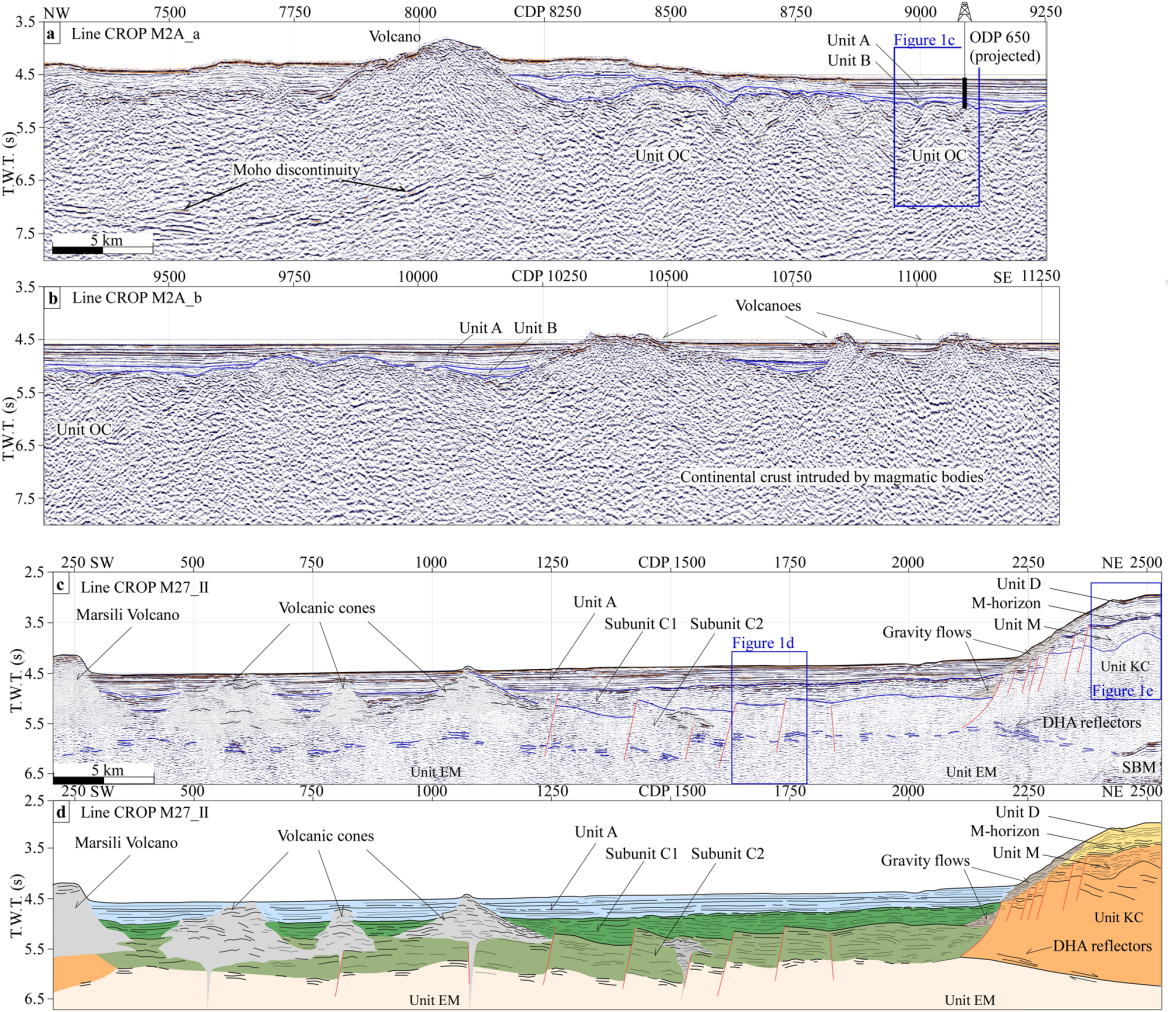
(**a** and **b**) Seismic line CROP M2A and its interpretation. (**c**) Seismic line Crop M27_II and (**d**) its line drawing interpretation. SBM: Sea-bottom multiple; T.W.T.: two‐way travel. See Fig. 1b for the location of the seismic lines.

The seismo-stratigraphic sequence I is recognised to the west of the Marsili volcano. It includes, from bottom to top, the units OC, B and A (Fig. 1c). Chaotic reflections, limited upward by a high-amplitude horizon, locally marked by hyperbolae diffractions characterise unit OC (Figs. 1c, 2a, b and Supplementary Fig. S1 online). Units B and A show continuous well-stratified reflectors with high frequency and medium to high-amplitude. Calibration of units OC, B and A with the stratigraphic succession sampled in the ODP Site 650 well-log indicates a correlation with the Island Arc Basalts of the oceanic crust unconformably overlain by limestone, dolomite to sandy mud deposits of ~ 1.87 to 0.45 Ma in age, topped by a graded sequence of gravel to sand-sized clastics with a low carbonate content of 0.45 Ma to Recent age^33^.

Seismo-stratigraphic sequence II is recognised to the east of the Marsili volcano (Fig. 1d). It includes from bottom to top, units EM, C and A. Unit EM shows sub-horizontal discontinuous reflections with moderate frequency and amplitude, bounded at the top by distinctive high-amplitude (DHA) reflectors (Fig. 1d, around CDP 2250 in Fig. 2c and d). We discuss the nature of unit EM in the next section.

Unit C overlies the DHA horizons. We have subdivided unit C into two subunits named C2 and C1 from bottom to top. Subunit C2 is characterised by layered reflectors with low- to moderate lateral continuity, high-frequency, and moderate- to high-amplitude (Fig. 1d). Reflections inside C2 are generally parallel to the DHA reflectors. Subunit C1 is defined by layered and continuous reflectors with high-frequency and -amplitude. It is marked at the top by a high‐amplitude and well‐defined laterally continuous event (Fig. 1d). In some regions, reflections diverge slightly toward the normal faults demonstrating syntectonic deposition. Unit A has the same characteristics described in the seismic-stratigraphic sequence I.

Seismo-stratigraphic sequence III is identified in the western termination of the Calabrian continental margin. It consists of three units named, from bottom to top, Unit KC, Unit M and Unit D (Fig. 1e). Unit KC is characterised by discontinuous reflections with variable amplitude and frequency underlying a high‐amplitude reflection of variable lateral continuity (tKC). Following our previous work^34^, we associate this seismic unit with the upper part of Kabilian‐Calabrian units.

Unit M is characterised by medium amplitude, discontinuous reflections. It is bounded at the top by a high‐amplitude, laterally continuous reflector (M horizon in Fig. 1e), a horizon of regional importance associated with the top of evaporites deposited during the late Messinian salinity crisis or to an erosional unconformity formed during the late Messinian sea-level fall^35^. Because of its stratigraphic position and seismic signature, we correlate the seismic facies of Unit M with the Oligocene (?) to lower Messinian clastic to terrigenous deposits that unconformably overly the Kabilian‐Calabrian units in the Calabrian Arc^36,37^.

Unit D overlies the M horizon. It shows layered medium- to high-lateral continuity, high-frequency, and moderate- to high‐amplitude reflectors. The sea-bottom limits upward Unit D (Fig. 1e). Based on the stratigraphic position, Unit D can be correlated with the Plio‐Quaternary sedimentary succession widespread in the Tyrrhenian Sea.

Chaotic reflections are recognised in the area close to the western termination of the Calabrian continental margin (around CDP 2250, Fig. 2c and d). They lie over units D and M and pinch out along the flank of the structural high that limits the Marsili Basin eastwards. We interpret these reflections as representative of gravity flow deposits.

Zones of chaotic reflectors with variable amplitude are detected around the Marsili volcano (CDPs 10200 to 11250 in Fig. 2b; CDPs 250–1200, around CDP 1500 in Fig. 2c and d;). They are characterised by a dome-shaped geometry, marked by hyperbolae diffractions, and limited upward by high-amplitude reflectors partly covered by a layer of sediment‐derived horizons. Based on their external and internal geometries, we interpret these zones as volcanic cones.

### Interpretation

The architecture of the Marsili Basin is reconstructed based on the seismo-stratigraphic sequences and tectonic features recognised on the seismic profiles.

The basalts of Unit OC are well-identified for ~ 50 km to the west of the Marsili volcano (Fig. 2a and b, CDPs 8850–9750). This oceanic field is bounded to the east by continental crust intruded by magmatic bodies and characterised by the occurrence of volcanoes (CDPs 10000–11250, Fig. 2b). On the map view, the latter appear as isolated cones or ridges located west and south of the Marsili volcano (Fig. 1b).

Westwards, the Unit OC is limited by a volcanic edifice. The latter is a part of the Pliocene volcanic arc (Fig. 1a) associated with the development of the Vavilov backarc basin^26^. Beneath the volcanic edifice, the Moho is imaged as a triplet of high-amplitude reflectors dipping towards the north-west at a depth between 6.2 and 6.8 s (Fig. 2a). Moving towards the Vavilov Basin, the Moho lies at a depth of ~ 7 s and show a sub-horizontal geometry.

Unit B deposits overlay the irregular top of Unit OC (Fig. 2a and b). Unit B reaches the maximum thickness of ~ 0.4 s in the westernmost sector of the Marsili basin. Unit A covers conformable unit B. Its maximum thickness is ~ 0.5 s. Deposits of units A and B are laterally continuous and undeformed.

In the eastern sector of the Marsili basin, unit EM is identified for ~ 30 km (Fig. 2c and d), and limited upwards by the DHA reflectors. The latter lie at a rough constant depth of ~ 6 s (CDPs 300–2100, Fig. 2d), whereas further northeast they dip towards the western Calabrian continental margin (around CDP 2250, Fig. 2d).

About 0.8 s thick deposits of subunit C2 cover unit EM and show onlap terminations of strata onto unit KC. West-dipping high-angle normal faults offset subunit C2 and the upper part of unit EM, bounding a series of tilted blocks, spaced ~ 4 km (CDPs 1000–1750, Fig. 2d). Deposits of subunit C1 unconformable overlay subunit C2, filling half-grabens formed above tilted blocks. Here, deposits show a fan-shaped geometry with strata inclined toward the normal faults, suggesting a syn-tectonic deposition (around CDP 1250, Fig. 2d). The thickness of Unit C1 is up to ~ 0.5 s. Strata of subnit C1 terminate with onlap geometry onto unit KC eastwards, and against the flanks of volcanic cones westward. Unit A overlays conformable subunit C1 with a maximum thickness of 0.45 s and shows onlap terminations onto the flanks of volcanic cones westwards.

A series of closely spaced high-angle normal faults offset deposits of units KC and M with displacements of ~ 0.25 s. Deposits of unit D unconformably overlay the unit M with a maximum thickness of 0.5 s. The lateral continuity of unit D is interrupted by gravity flow deposits that lie along the western flank of the structural high (around CDP 2250 in Fig. 2d).

Acoustic facies analysis shows that the seismo-stratigraphic sequences I and II are different except for Unit A (Fig. 1c and d). In particular, Unit OC, corresponding to basalt rocks, is not recognised to the east of the Marsili volcano. Here, sequence II shows seismic characteristics of reflectors for subunits C1 and C2 similar to those of the sedimentary cover of the unit KC, observed along the western Calabrian continental margin. Moreover, the pattern, frequency and amplitude of reflectors of unit EM are different from those observed for Unit OC (Fig. 1c and d). The top of unit EM lies between 5.8 and 6.1 s, whereas the top of unit OC reaches a depth of 5 s. Unit EM is overlaid by a ~ 1.2 s thick sedimentary cover (subunits C2 and C1). In contrast, the sedimentary cover of Unit OC reaches a maximum thickness of 0.4 s only (unit B in Figs. 1c; 2a and b). A series of high-angle normal faults offset subunit C2 deposits and the upper part of unit EM. Instead, any structure can be traced in the west sector of the Marsili Basin. Based on these differences, EM cannot be interpreted as oceanic crust. Prada et al. (2020)^38^ suggest that the basement of the eastern Marsili basin is made of exhumed mantle based on wide-angle seismic data. The average velocity-depth profile of the southeast Marsili basin fits the velocity-depth reference function for exhumed mantle regions of the Tyrrhenian and the Gulf of Cadiz^39^. The DHA reflectors, which limit upwards the unit EM, are interpreted as a shallow detachment surface in the western termination of the Calabrian continental margin (CDPs 2250–2500 Fig. 2d).

Mantle exhumation has been widely documented in the Mediterranean area during backarc extension (e.g. Gulf of Lion, Aegean Sea, Northern Tyrrhenian Sea)^21,23,40^. The age of the exhumation is difficult to constraint, but can be related either to the Mid-Miocene early phase of extension and exhumation well documented in Calabria^37,41,42^ or to the exhumation occurring in the Vavilov basin, where serpentinised harzburgite is overlaid by calc-alkaline basalts and fossiliferous sediments with an age of ~ 2.6 Ma^24,43^.

In summary, we propose that the Marsili basin consists of two sectors characterised by different nature of the basement, and roughly divided by the present-day Marsili volcano. The oceanic crust occurs in the western region, whereas exhumed mantle in the eastern one. The different nature of the basement to the east and west of the Marsili volcano is also supported by the density model of the Tyrrhenian region^44^, which highlight density values strongly compatible with an oceanic basin to the west of the Marsili volcano only.

Inspired by those results, we run a set of models to analyse the evolution of forearc and backarc basin during oceanic subduction. The numerical model parameters are not site-specific, in an attempt to remain generic and tackle the main tectonic processes and magmatism during retreating subduction.

### Numerical model of an ocean-continent subduction

A series of 2D petrological-thermo-mechanical models (Supplementary Table S1) was conducted simulating oceanic subduction beneath a continental plate. The numerical code i2vis^45^ uses finite differences and marker-in-cell methods, and handles viscous and plastic rheologies. The model incorporates effects that are essential for the study of subduction and upper plate deformation, such as sedimentation and erosion, hydration, and partial melting. Extracted melts are transmitted instantaneously to emplacement areas in the form of plutons and volcanics. It is assumed that 20% of all extracted melts propagate towards the surface, forming a volcanic arc above the extraction area^46,47^. The model domain is sized 4000 km × 720 km (Supplementary Fig.  S2 online). The rectangular grid of 1361 × 283 nodal points is non-uniform and contains a 1000 km wide and 200 km deep high-resolution area of 1 km × 1 km in the centre of the domain that gradually changes to 10 km × 10 km towards the model sides. Additional ca. 7.67 million markers are randomly distributed within the nodal points. The oceanic crust is represented by 3 km basalt and a further 5 km gabbroic crust. The thermal structure of the ocean is defined using a half-space cooling age of 50 Myr. The upper plate consists of an initial 20 km thick felsic crust of wet quartzite rheology underlain by a 15 km thick lower crust of plagioclase rheology. Both the lithospheric and the sub-lithospheric mantle are composed of anhydrous peridotite (dry olivine rheology). The initial thermal properties of the continents are horizontally uniform, and linearly increases from 0 °C on the surface and 675 °C at Moho depth and 1200 °C at 94 km depth. 0.5 °C /km gradient is defined in the underlying mantle. A 8 km wide weak zone defined by low plastic strength and wet olivine rheology is placed along the active margin to initiate subduction by a horizontal velocity condition of 2 cm/yr imposed on the downgoing plate (Supplementary Fig. S2 online). Our model results of arc rifting were inspired by previous inferences on subduction and forearc – backarc system modelling^2,46,48^.

**Figure 3:**
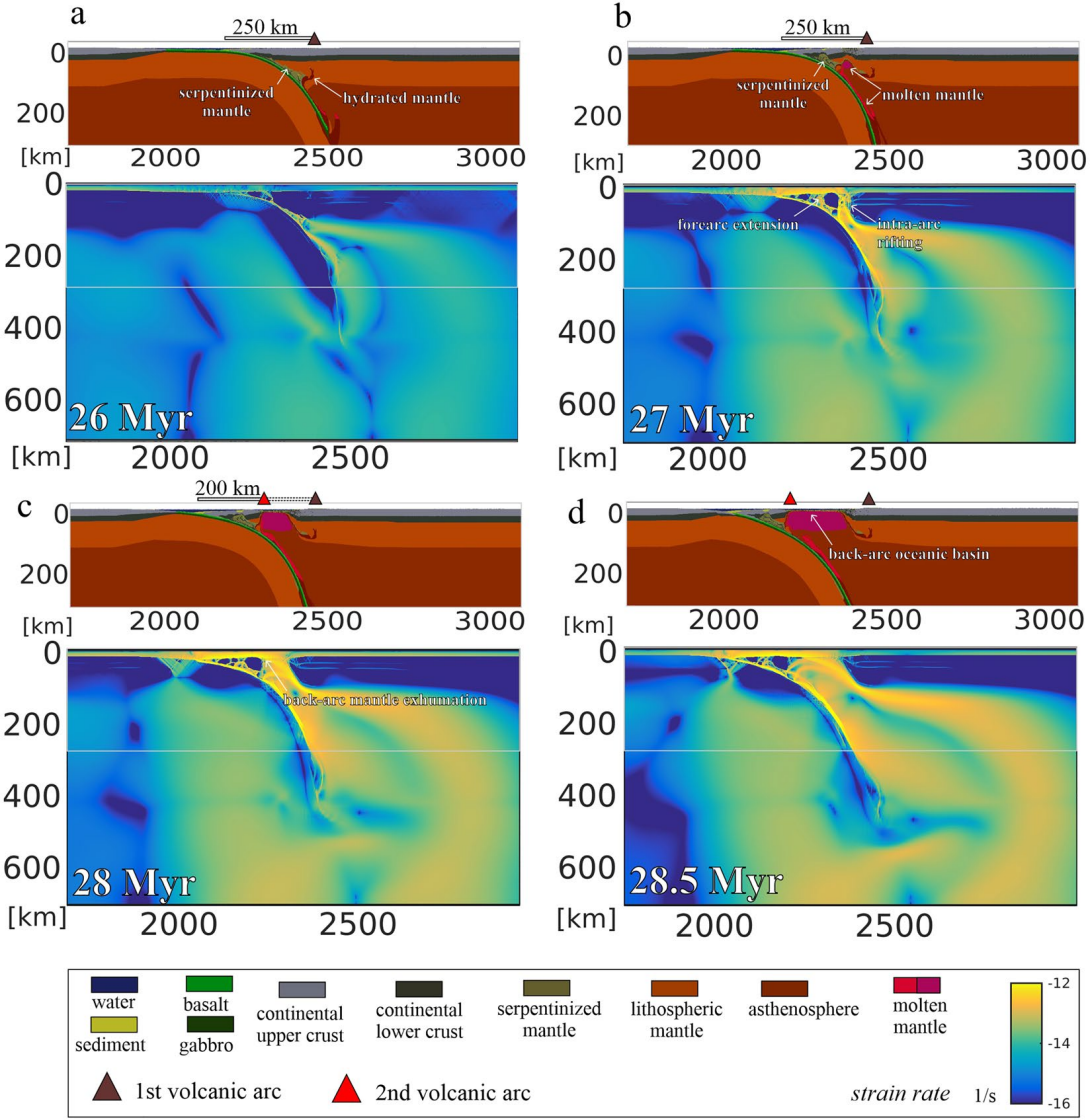
Thermo-mechanical model simulating the transition from forearc to backarc basin shown by strain rate results overlain by rock composition evolution. (**a**) Formation of a volcanic arc and forearc basin subsidence. (**b**) Arc rifting driven by melt induced weakening. (**c**) Slab retreat and backarc rifting. (**d**) Continued slab retreat and backarc basin formation in the previous forearc domain.

Subduction initiation and the early stages of subduction lead to the under-thrusting of the oceanic plate beneath the continental margin creating a subduction trench. Free-fall subduction is associated with the gradual consumption of the oceanic lithosphere and steepening of the slab. Fluids are released from the slab and hydrate the subduction interface and the overlying lithosphere. The presence of fluids also enables mantle serpentinisation. A first volcanic arc is formed overlying the hydrated mantle wedge initially at a 250 km distance from the accretionary wedge (Fig. 3a). This early stage of subduction corresponds with distributed normal faulting in the upper plate (Fig. 3a). After 26 Myr accelerated slab rollback results in fast trench retreat and related upper plate extension that is localised along the active volcanic arc (Fig. 3b), as it represents a rheological contrast due to the emplacement of volcanic and plutonic rock types. Melts are sourced from the asthenospheric upwelling from the hydrated mantle wedge as well as decompressional mantle melting occurs. Active normal faulting is localized along the active volcanic arc and within the forearc region (Fig. 3b). Stretching of the crust and lithospheric thinning also lead to the advective upwelling and exhumation of the previously serpentinised mantle in the close vicinity of the active volcanic arc (Fig. 3b). Continuous slab rollback causes extensional deformation and trench-ward migration of volcanism, leading to the formation of a second volcanic arc in the forearc domain (Figs. 3c). At this time contraction and thrusts are recorded within the accretionary wedge, normal faulting above the serpentinized forearc mantle and high deformation rate is shown along the active volcanic arc (Fig. 3c). By 28.5 Myr, the trench retreated ca. 180 km that was accommodated by the stretching and thinning of the previously formed volcanic arc and forearc domains (Fig. 3d). A backarc basin opens between the old and young volcanic arc, resulting in the decrease of the initial forearc region extension. The backarc basin shows only 2–3 km crustal thickness mainly infiltrated by melts and is overlain by young deepwater sediments. This scenario of forming the backarc basin from the former forearc domain is connected to melt induced rheological weakening^49^. In a series of models with lowered mantle potential temperatures melting is suppressed. In the absence of melt-induced weakening or assuming low fluid percolation velocities, no or only suppressed arc-rifting occurs (Supplementary Figs. 3 and 4 online).

## Discussion

**Figure 4:**
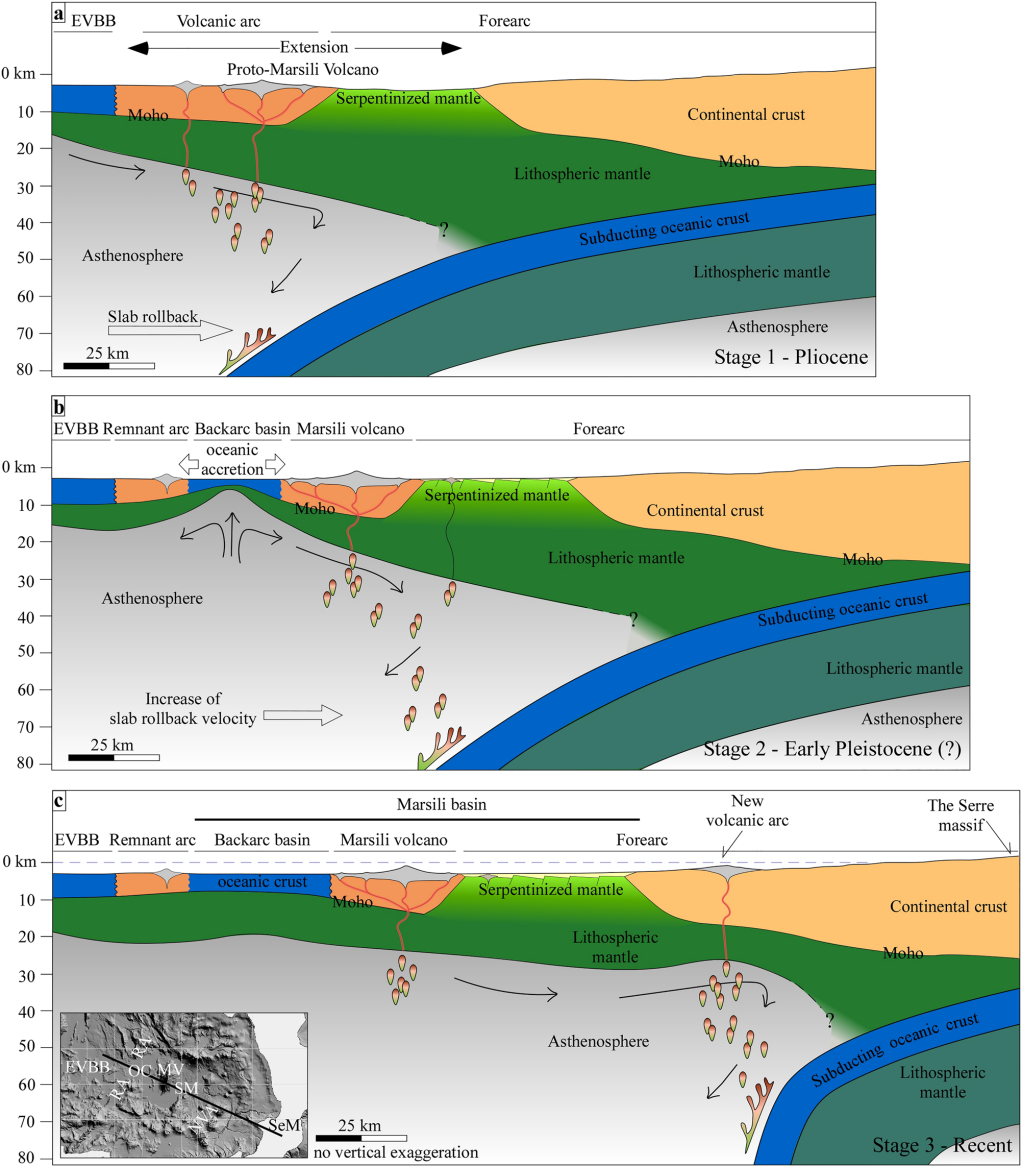
Evolutionary model of the Marsili basin. (**a**) Extensional deformation in the volcanic arc and forearc regions. (**b**) Arc rifting and oceanic accretion in the backarc basin. (**c**) Development of the new volcanic arc in the forearc domain. The depth of the Moho discontinuity and the lower plate derived from literature data^4,34,65–67^. Inset shows the location of the section. EVBB: eastern Vavilov backarc basin, MV: Marsili volcano, NVA: new volcanic arc, OC: oceanic crust, RA: remnant arc, SeM: Serre massif, SM: serpentinised mantle. The inset map was generated using the GeoSuite AllWorks software package (version 2021R2, https://www.geomarinesurveysystems.com/downloads/).

Figure 4 shows the evolution of the Marsili basin in three steps in agreement with the seismic data, and supported by the model results: the onset of the extensional deformation along the volcanic arc – forearc domains (Fig. 4a), the splitting of the volcanic arc, and the oceanic accretion in the backarc basin (Fig. 4b), and the formation of a new volcanic arc in the forearc region (Fig. 4c).

The first step (Fig. 4a) is balanced back removing the Marsili basin oceanic crust. At that time, the proto-Marsili volcano was probably part of the old (Pliocene) volcanic arc (central Tyrrhenian Sea, Fig. 1a), while backarc extension was localised in the Vavilov basin (EVBB in Fig. 4a). This scenario is based on the geometry of the Marsili volcano and its volcanic products. In fact, the comparison between the shape and the extension of the western flank of the Marsili volcano, and the central sector of the Pliocene volcanic arc, suggests that a smaller proto Marsili volcano was located along this old volcanic arc, to the north of Glauco/Garibaldi seamounts, before the oceanic accretion (Fig. 1b). This scenario also explains samples of medium-K calc-alkaline basalts and to a lesser extent of high-evolved K andesites^29^ recovered from the Marsili volcano, which may be well attributed to a volcanic arc on continental crust. The proposed scenario justifies the actual position of the Marsili volcano with respect to the oceanic basin because it is the only volcanic ridge ahead of the oceanic sector of the Marsili Basin. In fact, the Aeolian Islands (1 Ma – recent) cannot represent the volcanic arc of the Marsili backarc basin because they are younger than the oceanic crust (1.87 Ma).

In the first stage, exhumed mantle lies to the east of the volcanic arc (Fig. 4a). As discussed before, the timing of the mantle exhumation remains an open question. Based on our numerical modelling, the acceleration of the slab rollback leads to extensional deformation along the volcanic arc and exhumation of the serpentinised mantle in the forearc domain close to the volcanic arc (Fig. 3b). Therefore, it is probable that the mantle exhumation occurred before the oceanic crust formation in the Marsili basin. This timing agrees with the thicker sedimentary cover overlaying the exhumed mantle compared to the one above the oceanic crust, and with the activation of detachment fault described in the Calabrian onshore^37^ or with the exhumation occurring in the Vavilov basin^24^.

Continuous stretching of the lithosphere beneath the volcanic arc and forearc domain caused the formation of several normal faults dissecting the exhumed mantle (Figs. 2d and 4b), and led the volcanic arc region to break up. Due to the arc rifting, a part of the volcanic arc, corresponding to the proto-Marsili volcano, shifted towards the southeast, while the remnant side was left behind and remained fixed (Fig. 4b). So, an oceanic backarc basin started forming between the Marsili volcano and the remnant volcanic arc.

The continental break-up in correspondence of the volcanic arc can be explained considering that this region represents the weakest portion of the arc-forearc domain^50^ due to the emplacement of volcanic and plutonic rocks. This scenario is supported by the numerical modelling where extensional deformation is first localised in the volcanic arc region due to fluids that create weakness of the lithosphere generating a rheological contrast between the volcanic rocks and the surroundings.

Once backarc spreading started, the slab rollback increased the velocity substantially^7,51^. Accordingly, the lower plate migrated towards the southeast in the last stage of the model (Fig. 4c). The partial melting of the asthenosphere and the magma rising occurred beneath the forearc region, and here caused the formation of a new volcanic arc (Fig. 4c), corresponding to the Aeolian Arc (AA in Fig. 1a).

The development of a new volcanic arc in the forearc region marks the onset of a new cycle for forming a new backarc basin at the expense of the forearc domain, as our numerical modelling documents (Fig. 3c and d). The transition from forearc to backarc region is not completed in the forearc domain to the east of the Marsili volcano. If the rollback continues, a backarc basin will develop between the Marsili volcano and the Aeolian Islands.

In Recent time, the Marsili volcano continued to be fed as suggested by the summit andesitic lavas aged at 0.1–0.2 Ma^52^ and characterised by Island Arc Basalt (IAB) affinities, similar to those of the Aeolian arc^29^. Thus, the vertical accretion of the Marsili edifice volcano results from three phases of activity: during the Pliocene, it was a part of the volcanic arc associated with the development of the Vavilov backarc basin; successively, it became the volcanic arc related to the formation of the Marsili backarc basin, and in Recent time, it is fed together with the Aeolian Islands. This interpretation fits with the elevated magma supply (volume on the order of 1500 km^3^) documented for the Marsili volcano^32^.

## Conclusions

Our results highlight that the Marsili volcano, commonly interpreted as the spreading centre of the Marsili backarc basin, was instead a part of an old (Pliocene) volcanic arc that was successively affected by arc rifting. The shift of the Marsili volcano eastwards led to the formation of an oceanic backarc basin located between the Marsili volcano and the old remnant arc, which remained fixed. The eastern side of the Marsili basin, previously considered as the other half of the oceanic backarc basin, is instead a part of the forearc domain floored by serpentinised mantle. As slab rollback continued, volcanism migrated towards the trench and a new volcanic arc (Aeolian Island) formed in the forearc domain. The formation of the new volcanic arc represents the onset of the forearc-rifting that may be lead to the opening of a backarc basin between the old and young volcanic arc, resulting in the decrease of the initial forearc region extension.

The example of the Tyrrhenian Sea illustrates how the evolution of forearc and backarc domains is intimately interconnected. Fluids, released from the downgoing plates, control lithospheric hydration and mantle serpentinisation as well as asthenospheric mantle melting. Fluids and melts induce weakening in the upper plate that drives the transition from forearc subsidence through arc-rifting to backarc spreading accompanied by migration of the volcanic arc.

## Methods

### Seismic reflection profiles

We use 160 km of multi-channel, high-penetration reflection seismic profiles (Crop M27 and Crop-M2A lines, see Scrocca et al., 2003 for detailed acquisition parameters)^53^ and ca. 30 km of high-resolution, multi-channel, seismic reflection profile (Marsili 1 line, see Pepe et al., 2000 for detailed acquisition parameters)^54^. Pre-stack data processing includes: sorting of the Common Depth Point (CDP), velocity analysis on every 50 CDP, normal move out, and stack of the CDP. The used data set was clipped at 7.5 s (two-way travel time, T.W.T.). Post-stack data processing includes: (a) true amplitude recovery using a T^2^ spherical divergence correction; (b) band-pass (30–200 Hz) “Finite Impulse Response” filter using a filter length of 256 samples; (c) de-ghosting, (d) Kirchhoff time migration; (e) trace mixing of three traces for enhancing horizontal signal; (f) time-variant gain to boost amplitudes of deeper arrivals; (g) mutes to eliminate the signal noise on the water column. For imaging of the oceanic crust event, we used similar processing steps, with the exception of the migration step. After all processing was completed, the CROP M2A and Marsili 1 lines had two processed versions: a time migrated section for accurate seafloor and basement interfaces (Fig. 2a and b) and a velocity stack for the Oceanic Crust event (see supplementary Fig. S1 online). We interpreted and laterally mapped the top of the oceanic crust from the time migrated sections.

### Numerical modelling

This study used i2vis^45^, which solves the mass, momentum, and energy conservation equations on a Eularian staggered grid, using finite differences and marker-in-cell techniques. Conservation of mass is described by the Lagrangian continuity equation for a compressible fluid:

$$\frac{{\partial v_{{\text{x}}} }}{{\partial {\text{x}}}} + \frac{{\partial v_{{\text{y}}} }}{{\partial {\text{y}}}} = { - }\frac{{1}}{\rho }\frac{D\rho }{{Dt}},$$

where $\frac{D\rho }{{Dt}}$ is the substantive time derivative of density, which accounts locally for melt extraction.

The momentum Eq. (2D Stokes) takes the form:

$$\frac{{\partial \sigma^{\prime}_{xx} }}{\partial x} + \frac{{\partial \sigma_{xy}^{{}} }}{\partial y} = \frac{\partial P}{{\partial x}},$$

$$\frac{{\partial \sigma_{xy} }}{\partial x} + \frac{{\partial \sigma^{\prime}_{yy} }}{\partial y} = \frac{\partial P}{{\partial y}} - g\;\rho \left( {{\text{T}},{\text{ P}},{\text{ C}}} \right),$$

where $\sigma^{\prime}_{xx}$, $\sigma_{xy}$, $\sigma^{\prime}_{yy}$ are deviatoric stress tensor components. The density $\rho$(T, P, C) depends on temperature (T), pressure (P) and composition (C) and $g$ is the acceleration due to gravity.

The heat conversion equation is solved on a Lagrangian frame^45^:

$$\rho C_{p} \left( {\frac{DT}{{Dt}}} \right) = - \frac{{\partial q_{x} }}{\partial x} - \frac{{\partial q_{y} }}{\partial y} + H_{r} + H_{a} + H_{s} + Q_{l} ,$$

$$\begin{gathered} q_{x} = - k{\text{(P, T, C)}}\frac{\partial T}{{\partial x}}, \hfill \\ q_{y} = - k{\text{(P, T, C)}}\frac{\partial T}{{\partial y}}, \hfill \\ \end{gathered}$$

where $$\frac{DT}{{Dt}}$$ is the substantive time derivative of temperature, $$C_{p}$$ is the isobaric heat capacity; $$q_{x}$$ and $$q_{y}$$ are heat fluxes; $$k$$$${\text{(P, T, C)}}$$ is the thermal conductivity, which is a function of pressure, temperature and composition; $$H_{r}$$,$$H_{a}$$, $$H_{s}$$ are the radiogenic, adiabatic and shear heat components, respectively.

$H_{r}$ = constant for a given rock composition

$$H_{a} = T\alpha \frac{DP}{{Dt}},$$

$$H_{s} = \sigma^{\prime}_{xx} \dot{\varepsilon }_{xx} + \sigma^{\prime}_{yy} \dot{\varepsilon }_{yy} + 2\sigma^{\prime}_{xy} \dot{\varepsilon }_{xy},$$

where $\alpha$ is the thermal expansion coefficient and $\dot{\varepsilon }_{xx}$,$\dot{\varepsilon }_{yy}$,$\dot{\varepsilon }_{xy}$ are components of the deviatoric strain rate tensor.

The effect of latent heating/cooling $Q_{\text{l}}$ due to equilibrium crystallisation/ melting is included implicitly by increasing the effective heat capacity $C_{Peff}$ and the thermal expansion $\upalpha_{eff}$ of melting/crystallising rocks:

$$\begin{gathered} C_{{p_{eff} }} = C_{p} + Q_{l} \left( {\frac{\partial M}{{\partial T}}} \right)_{P = const} \hfill \\ \alpha_{eff} = \alpha + \rho \frac{{Q_{l} }}{T}\left( {\frac{\partial M}{{\partial P}}} \right)_{T = const} \hfill \\ \end{gathered}$$

$Q_{\text{l}}$ is the latent heat of rock melting.

All boundary conditions are free slip. The top surface of the oceanic and continental crust is treated as an internal free surface by using a low viscosity (10^18^ Pa s) and low density (1 kg/m^3^ for air, 1000 kg/m^3^ for water) top layer (initially 10 km). The interface between this weak layer and the top of the oceanic/continental crust evolves spontaneously. Further on, the topography of the model (air/crust interface) evolves according to a transport equation. It accounts for sedimentation and erosion and is calculated for each time-step^55^:

$$\frac{{\partial y_{{{\text{es}}}} }}{{\partial {\text{t}}}} = v_{{\text{y}}} - v_{{\text{x}}} \frac{{\partial y_{{{\text{es}}}} }}{{\partial {\text{x}}}} - v_{{\text{s}}} + v_{{\text{e}}} = {0,}$$

where $y_{{{\text{es}}}}$ is the vertical position of the surface as a function of the horizontal distance x: $v_{{\text{y}}}$ and $v_{{\text{x}}}$ describe the vertical and horizontal components of the material velocity vector at the surface. $v_{{\text{s}}}$ and $v_{{\text{x}}}$ are sedimentation and erosion rates, respectively.

All rheologies are visco-plastic. Viscous (ductile) deformation is computed as a combination of dislocation and diffusion creep and depends on temperature, pressure and strain rate. A smooth transition between dislocation creep and diffusion creep is assumed to occur at 10^4^ Pa^56^. The viscosity for dislocation creep is defined as follows:

$${\eta _{creep}} = \frac{{\dot \varepsilon _{II}^{\frac{{1 - n}}{n}}}}{{\mathop {A_D^{}}\nolimits^{\frac{1}{n}} }}\exp \left( {\frac{{Ea + PVa}}{{nRT}}} \right),$$

where ${\dot \varepsilon _{II}} = \sqrt {{1 \mathord{\left/ {\vphantom {1 2}} \right. \kern-\nulldelimiterspace} 2}{{\dot \varepsilon }_{ij}}{{\dot \varepsilon }_{ij}}}$ is the second invariant of the strain rate tensor. The terms *A*_*D*_ (pre-exponential factor), *Ea* (activation energy), *Va* (activation volume) and *n* (creep exponent) are experimentally determined flow law parameters. R is the gas constant.

Plasticity (brittle failure) is implemented using a yield criterion, limiting the creep viscosity,$\eta_{creep.}$ Additionally, we assume that fluid and melt lower the plastic strength of rocks in our model. The yield strength is decreased according to the prescribed pore fluid and melt pressure factors, $\lambda_{fluid}$ and $\lambda_{melt}$_,_ respectively.

$$\eta_{creep} \le \sigma_{yield} /2\mathop {\varepsilon_{II} }\limits^{.} ,$$

$$\sigma_{yield} = c + P\sin (\phi ),$$

$$\sin (\varphi ) = \sin (\phi dry)\lambda_{fluid/melt} ,$$

$$\lambda_{fluid} = 1 - P_{fluid} /P_{solid} ,$$

$$\lambda_{melt} = 1 - P_{melt} /P_{solid} ,$$

where *c* is the cohesion, which is the strength at *P* = 0 and effective internal friction angle (*φ*) calculated from the friction angle of dry rocks, $\phi dry$. The terms *P*_*solid*_ and *P*_*fluid/melt*_ designate dynamic pressure and pore fluid/melt pressure, respectively.

In order to simulate mineralogical reactions such as mineralogical phase changes, melting reactions, fluid release and consumption, we used coupled petrological-thermomechanical numerical modelling approach^45,46^. According to this approach, stable phase relations for different lithologies are computed for Lagrangian rock markers at every time step based on minimisation of Gibbs free energy^57^ as a function of local pressure, temperature and rock composition. For this purpose, thermodynamic properties of fluids, melts and minerals are obtained from internally consistent thermodynamic database^45,58^. All these properties are used to pre-compute look up tables for each lithology with a resolution of 5 K and 25 MPa for T and P, respectively. Subsequently, these tables are used in a thermomechanical experiment to compute certain in-situ properties, for example water content for a large amount of markers. The examples of phase diagrams computed for different lithologies can be found in previous studies^59^. At every time step, these pre-computed properties are updated for all Lagrangian markers.

The model assumes that pore water content decreases due to compaction from 2 wt.% to 0 wt.% at a depth of 75 km:

$$x(wt.\% ) = (1 - 0.013\Delta y)X_{{H_{2} O(p0)}},$$

where $X_{{H_{2} O(p0)}}$ = 2 wt.% is the free water content at the surface and Δ*y* is the depth in km below the surface (0–75 km). The time and amount of water released by dehydration reactions are determined by the assumption of thermodynamic equilibrium and the model of physicochemical conditions^60–62^. The stable water content for different lithologies is calculated based on Gibbs free energy minimization^57^ as a function of pressure and temperature^45^. Further on, expelled water moves upwards instantaneously until it reaches rock which assimilates an additional amount of water. This process is computed according to pressure gradients^63^ as:

$$\begin{gathered} v_{x(water)} = v_{x} - A\left( {\frac{\partial P}{{\partial x}}} \right) \hfill \\ \hfill \\ \end{gathered}$$

$$v_{y(water)} = v_{y} - A\left( {\frac{\partial P}{{\partial y}} - \rho_{fluid} g} \right)$$

$$A = \frac{{v_{percolation} }}{{g\left( {\rho_{mantle} - \rho_{fluid} } \right)}}$$

*v*_*x(water)*_ and *v*_*y(water)*_ are the fluid velocities in x and y direction *v*_*x*_ and *v*_*y*_ indicate the local velocity of the solid mantle, *A* is a water percolation constant, $v_{percolation} = 10$ cm/yr, a presumed standard water percolation velocity^60,64^, g = 9.81 m/s is the gravitational acceleration, ρ_*mantle*_ = 3300 kg/m^3^ and ρ_*fluid*_ = 1000 kg/m^3^ is the density of the mantle and fluid, respectively.

Following previous studies, the water transport model does not permit complete hydration of the peridotitic mantle^61^. Therefore, the hydrated mantle solidus is located between the wet and dry peridotite solidi. Both hydrous and dry melting increases linearly with temperature and pressure^45^. The volumetric amount of melt, M_0_, for a given pressure and rock composition is based on:

$$M_{0} = 0$$ when $$T < T_{solidus}$$,

$$M_{o} = \left( {T - T_{solidus} } \right)/\left( {T_{liquidus} - T_{solidus} } \right)$$ when $$T_{solidus} < T < T_{liquidus}$$,

$$M_{0} = 1$$ when $$T > T_{liquidus}$$.

T_solidus_ is the solidus temperature where wet and dry solidi are used for the hydrated and dry mantle. T_liquidus_ represents the dry liquidus temperature at a given pressure and rock composition. Melt extraction from partially molten rocks occurs where the melt extraction exceeds a pre-defined melt threshold of M_max_ = 4%. A non-extractable amount of melt M_min =_ 2% remains in the source^46,62^. The total amount of melt, M, for every marker considers the amount of previously extracted melt and is calculated as:

$$M = M_{0} - \sum\nolimits_{n} {M_{ext} } ,$$

where $\sum\nolimits_{n} {M_{ext} }$ is the total melt fraction extracted during previous *n* extraction episodes. Rocks are considered refractory when the extracted melt fraction is larger than the standard one (i.e. when $\sum\nolimits_{n} {M_{ext} } > M_{0}$). When the total amount of melt *M* exceeds the threshold *M*_*max*_, the melt fraction *M*_*ext*_ = *M – M*_*min*_ is extracted and is $M_{ext}$ updated. Extracted melts are transported instantaneously to the near-surface environment. Here they form volcanic arcs (20%) or emplace within the continental crust (80%) at levels of the highest possible intrusion rate (i.e. highest possible local crustal divergence rate, *div*_*crust*_). The emplacement level is calculated by evaluating the ratio of the effective melt overpressure and the effective viscosity of the crust above the extraction region:

$$div_{crust} = \left[ {Py_{melt} - g_{y} \rho_{melt} \left( {y_{melt} - y} \right) - Py} \right]/\eta_{y} ,$$

where *Py*_*melt*_ is the pressure at the extraction level *y*_*melt*_ and *Py* is the pressure at the current level *y*, *g* is gravitational acceleration in y-direction [m/s^2^], *ρ*_*melt*_ is the melt density and *η*_*y*_ is the effective local crustal viscosity at the current level y.

The effective density*, ρ*_*eff*_*,* of partially molten rocks is defined by:

$$\rho_{eff} = \rho_{solid} - M(\rho_{solid} - \rho_{melt} ),$$

where $\rho_{solid}$ and $$\rho_{\begin{subarray}{l} melt \\ \end{subarray} }$$ are the standard densities of solid and molten rocks, respectively and $\rho P,T$ is the density of solid rocks at given *P*(MPa) and *T*(K) computed from:

$$\rho_{P,T} = \rho_{0} \left[ {1 - \alpha \left( {T - T_{0} } \right)} \right]\left[ {1 + \beta \left( {P - P_{0} } \right)} \right]$$

where $\alpha$ and $\beta$ denote the thermal expansion and compressibility coefficients, respectively.

## Supplementary Information

Supplementary Information.
