# Supplementary material for: Arc and forearc rifting in the Tyrrhenian subduction system
Source: Sci Rep. 2022 Mar 18;12:4728. doi: 10.1038/s41598-022-08562-w (PMC8933539; doi:10.1038/s41598-022-08562-w)
Supplement: Supplementary file 1 — Supplementary Information. [file 41598_2022_8562_MOESM1_ESM.pdf]

Arc and forearc rifting in the Tyrrhenian subduction system

Corradino M., Balazs A., Faccenna C., Pepe F.

Supplementary material: Figures S1, S2, S3, S4, S5, S6, S7, Table S1

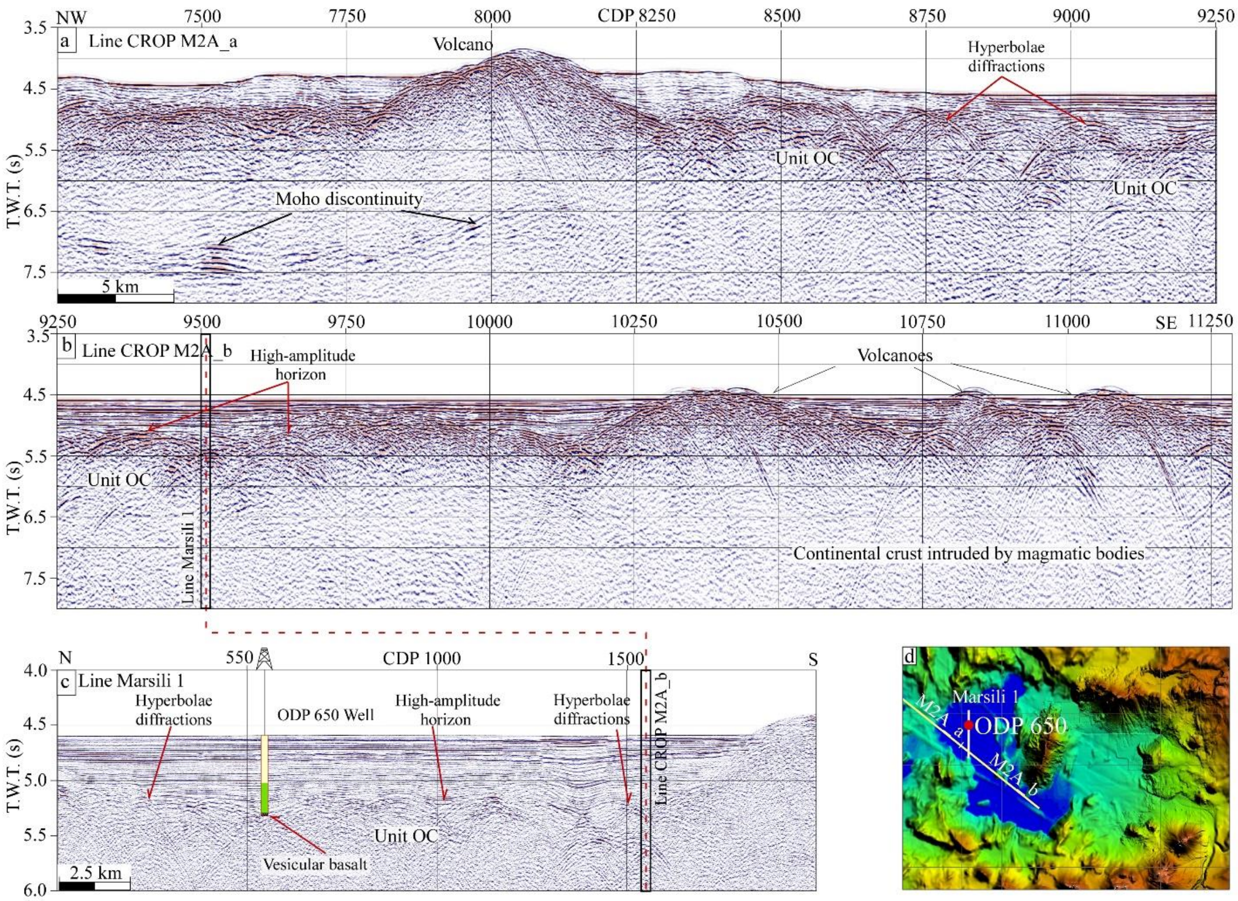

Supplementary Figure S1. Unmigrated version of the multichannel seismic reflection profiles (a and b) CROP M2A and (c) Marsili 1, showing as much as 500 ms of well-layered, flat-lying sedimentary fill with close-spaced, laterally continuous, variable-amplitude internal reflectors that overlies an irregular, strongly diffracting seismic unit (Unit OC). In box c, the ODP 650 Well shows that the top of Unit OC is composed of vesicular basalts. We interpret Unit OC as representative of the rough top of the oceanic crust emplaced in the western sector of the Marsili Basin, based on the well data and diffractive nature of the Unit OC. CDP: common depth point; T.W.T.: two-way travel. (d) Seismic profiles location. The map was generated using the GeoSuite AllWorks software (version 2021R2, https://www.geomarinesurveysystems.com/downloads/).

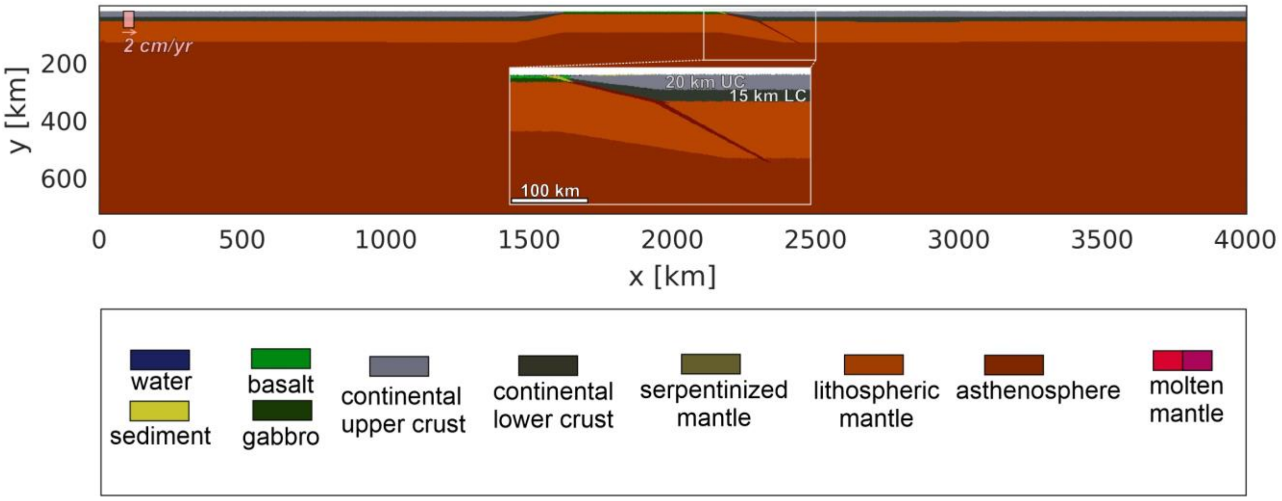

Supplementary Figure S2. Initial compositional setting of the numerical model. All boundaries are free slip and the lower plate is pushed by an internal boundary condition of 2 cm/yr. 16 km thick low density (1000 kg/m^3), low viscosity (10^{18} Pa s) sticky air layer is defined on top of the model. The thermal structure of the ocean is defined using a half-space cooling age of 50 Myr. The initial thermal properties of the continents are horizontally uniform, 0 ◦C on the surface and 675 ◦C at Moho depth and assume 8 ◦C /km and 0.5 ◦C /km gradients in the lithospheric and asthenospheric mantle, respectively. The temperature at the bottom of the model at 720 km is 1604 ◦C. Internal velocity boundary condition is defined close to the left model boundary (pink rectangle).

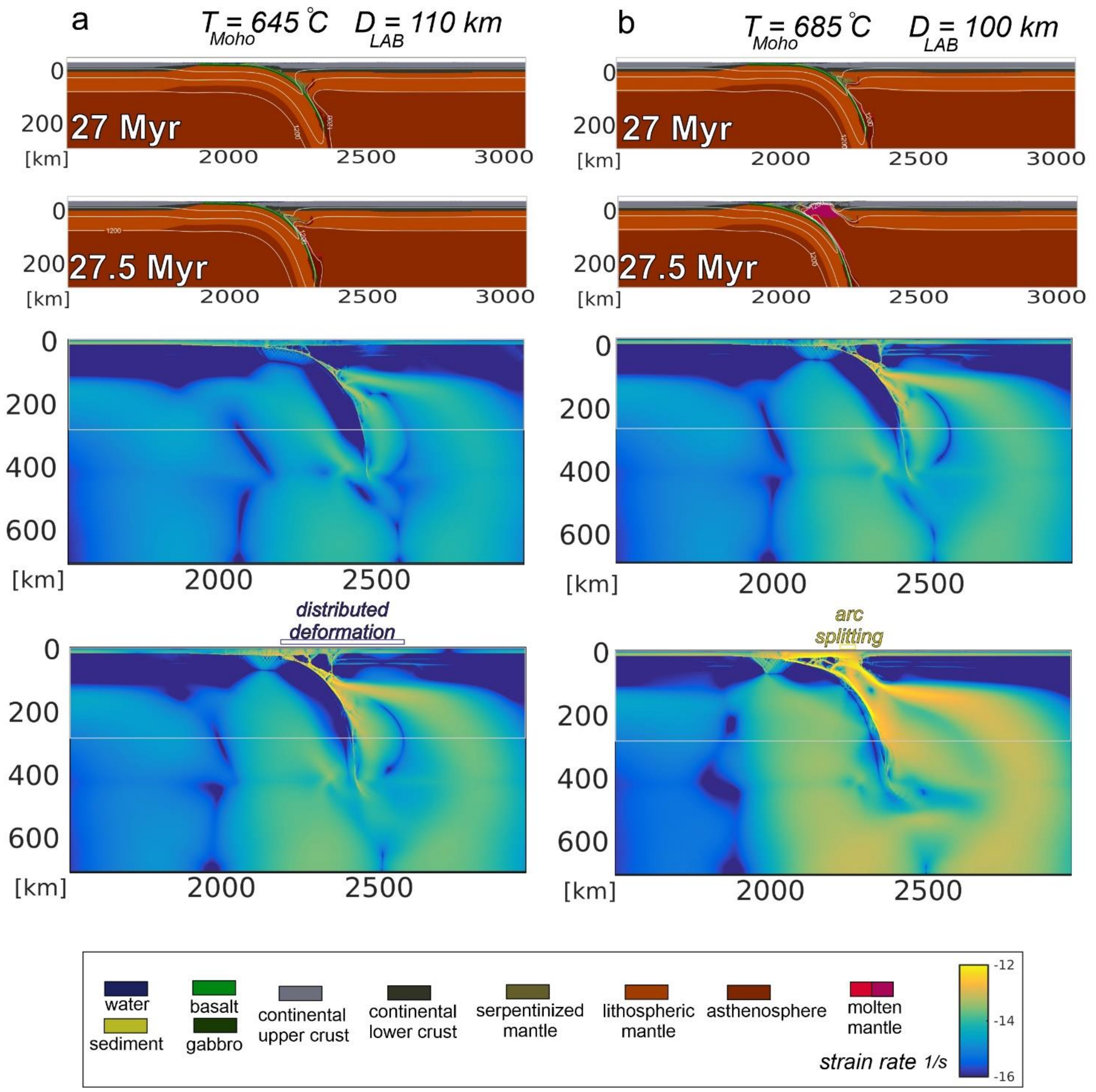

Supplementary Figure S3. Numerical model results assuming different Moho temperatures and lithospheric thicknesses of the upper plate. (a) Lower upper plate thermal gradients lead to suppressed mantle melting resulting in distributed extensional deformation. (b) Higher upper plate thermal gradients lead to earlier mantle melting, weakening and promote earlier arc rifting.

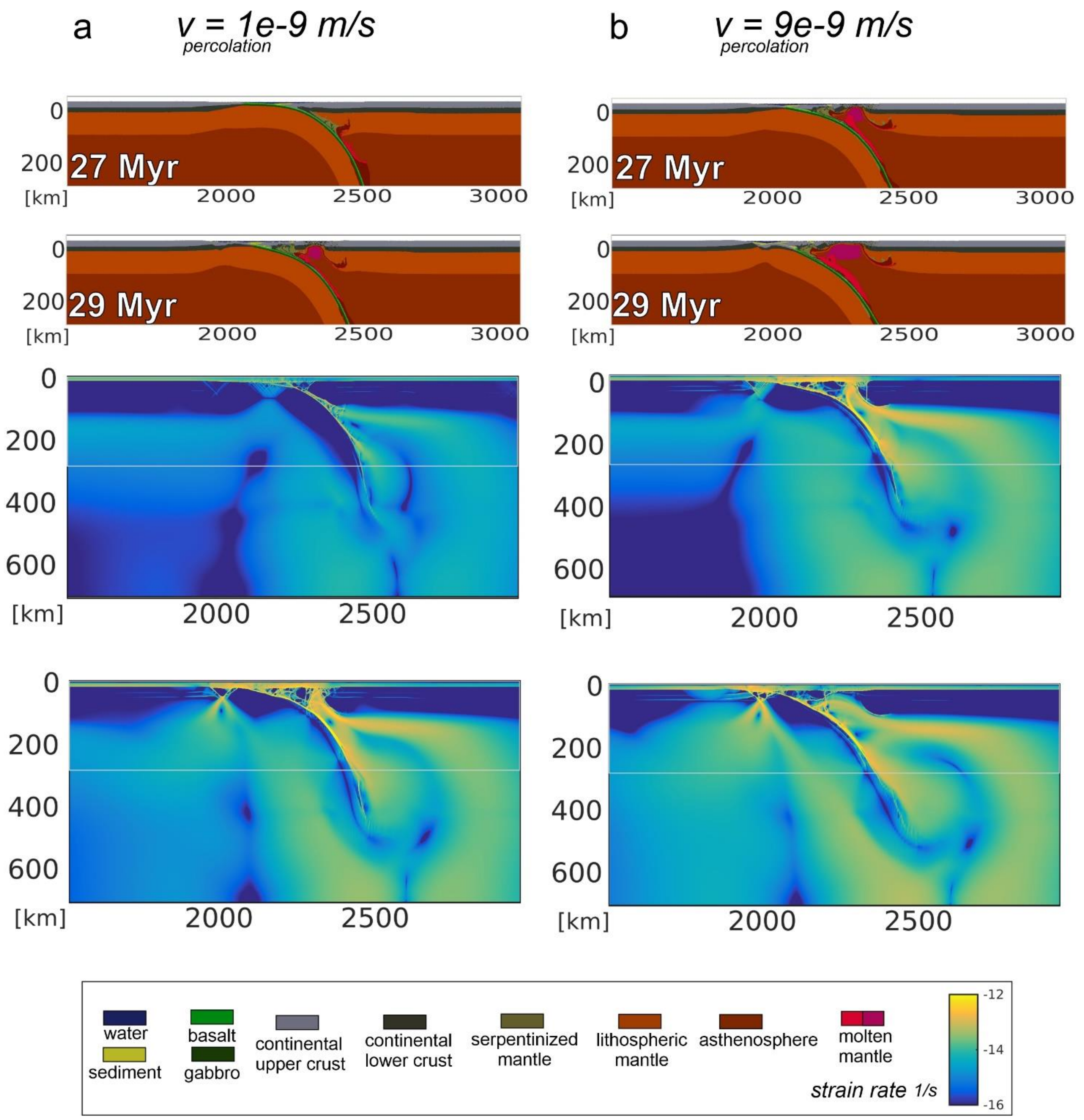

Supplementary Figure S4. Numerical model results of subduction and arc rifting with different fluid percolation velocities. Fluids are released from the subducted basalt and sediments, and propagate towards the overlying mantle wedge. A higher fluid percolation velocity leads to earlier hydration and mantle weakening and promotes earlier arc rifting.

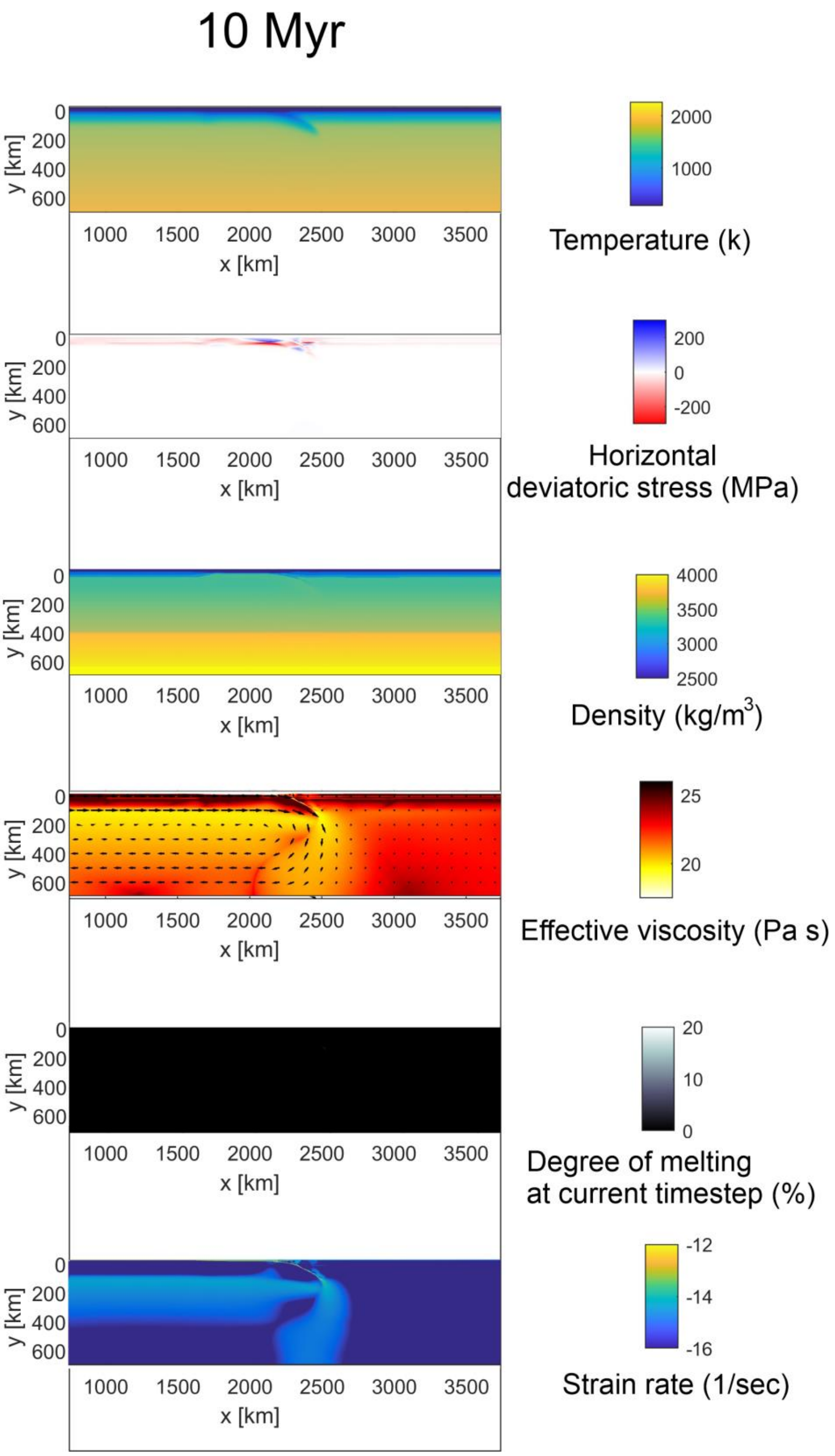

Supplementary Figure S5: Maps of temperature, horizontal component of the deviatoric stress field, density, effective viscosity, degree of melting, and strain rate after 10 Myr of evolution.

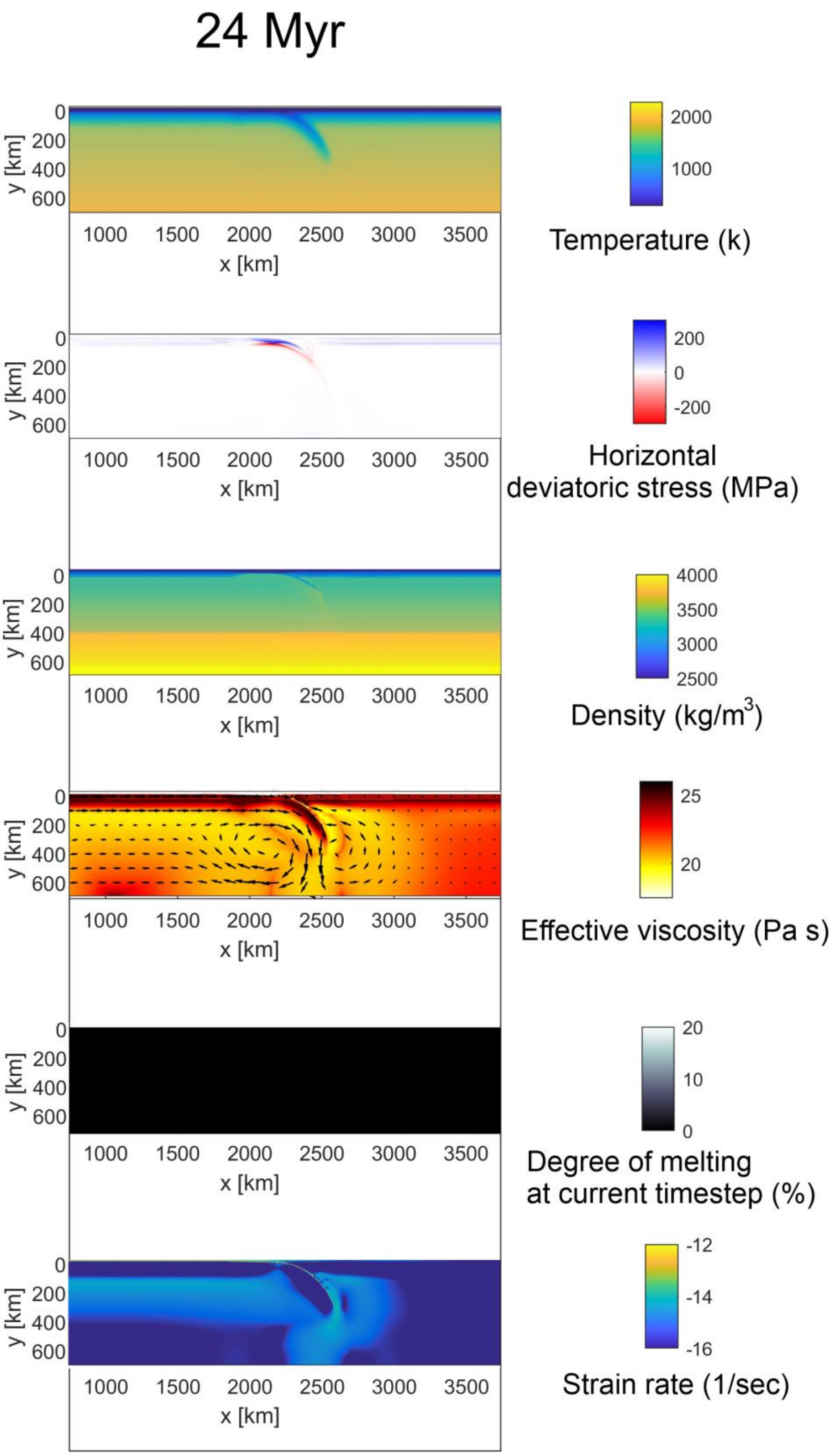

Supplementary Figure S6: Maps of temperature, horizontal component of the deviatoric stress field, density, effective viscosity, degree of melting, and strain rate after 24 Myr of evolution.

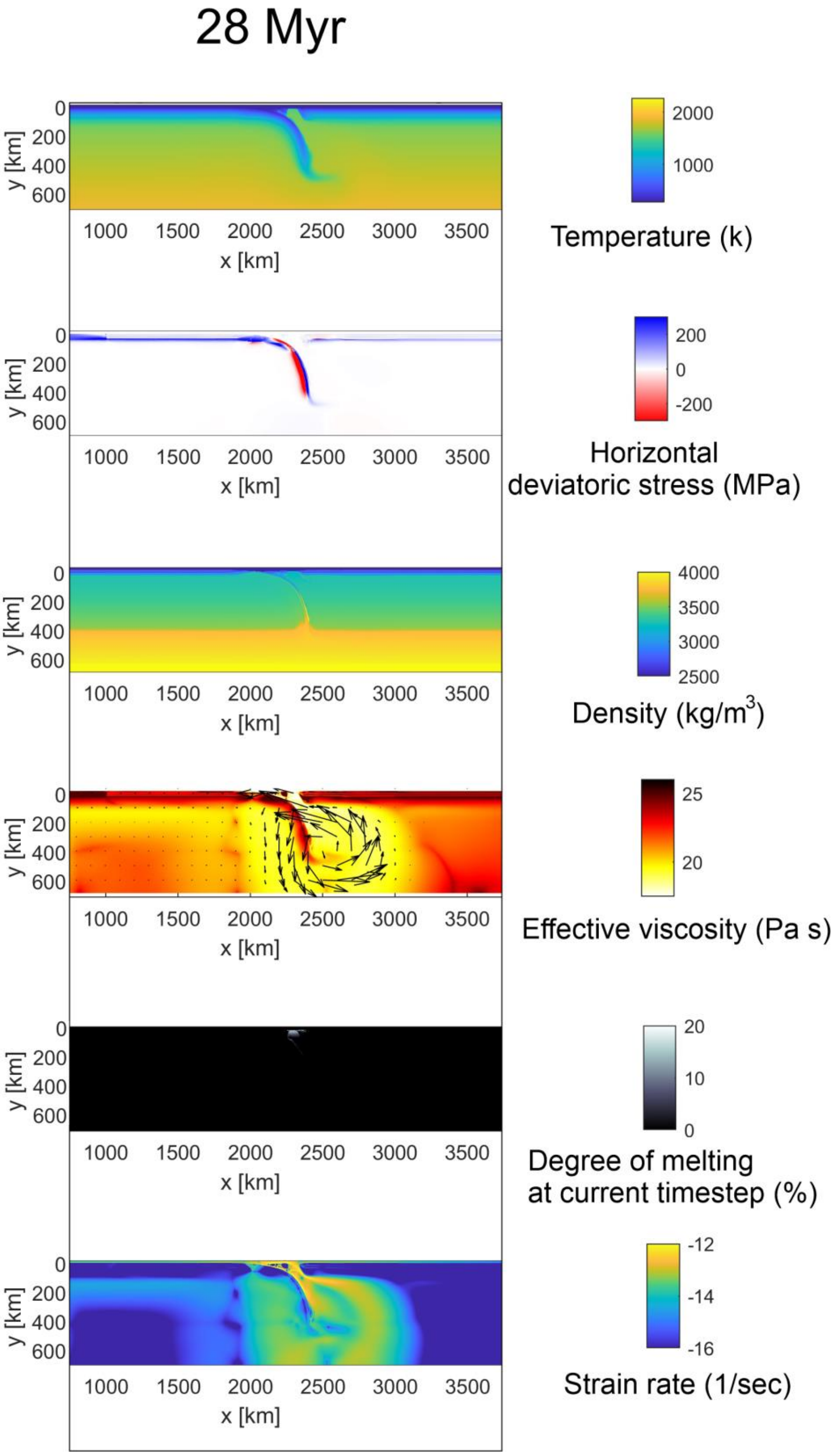

Supplementary Figure S7: Maps of temperature, horizontal component of the deviatoric stress field, density, effective viscosity, degree of melting, and strain rate after 28 Myr of evolution

	Upper cont. crust	Lower cont. crust	Lithospheric mantle	Basalt	Gabbro	Weak zone	Sediments
Thickness (km)	20	15	75	3	5	-	varies
Rheology	wet quartzite	plagioclase	dry olivine	plagioclase	plagioclase	wet olivine	wet quartzite
Density, ρ_0 (kg m^{-3})	2750	3000	3300	3000	3000	3200	2600
Pre-exponential factor, $1/A_D$ (Pa^n s)	1.97×10^{17}	4.8×10^{22}	3.98×10^{16}	4.8×10^{22}	4.8×10^{22}	5.0×10^{20}	1.97×10^{17}
Activation energy, E (kJ mol^{-1})	154	238	532	238	238	470	154
Power law exponent, n	2.3	3.2	3.5	3.2	3.2	4.0	2.3
Cohesion (Pa)	$3\text{-}2 \times 10^6$	3×10^6	3×10^6	3×10^6	3×10^6	$2\text{-}1 \times 10^6$	3×10^6
Coefficient of friction, sin(φ)	0.15-0.10	0.10	0.30	0.125	0.30-0.10	0.60-0	0.100
Radioactive heat production, H_r (μW/m^3)	2	0.200	0.022	0.022	0.024	0.022	2

Supplementary Table S1. Main parameters of the numerical models. The rheological rock properties are adapted from previous similar studies (e.g., Gerya and Yuen, 2007; Gerya and Meilick, 2011; Vogt et al., 2012). Plastic strain weakening is applied between 0.0 and 0.5 strain values.
